# Rare Variants of Carotid-Vertebrobasilar Anastomoses

**DOI:** 10.5334/jbr-btr.1167

**Published:** 2016-08-31

**Authors:** Bulent Petik, Deniz Colak, Mehmet Sirik, Sukru Mehmet Erturk

**Affiliations:** 1 Adiyaman University Medicine Faculty, TR

**Keywords:** Carotid, Vertebrobasilar anastomoses, Trigeminal artery, Hypoglossal artery, Persistent otic artery

## Abstract

Carotid-vertebrobasilar anastomoses generally disappear during embryogenesis. However, if a problem exists during regression, these arteries persist in adult period and are named as persistent arteries. Their persistence in adult patients is sometimes pathological and may result in the development of an aneurysm or a compressive syndrome. These anastomoses are frequently associated with proximal or distal arterial pathology. Herein, we present three rare variants of carotid-vertebrobasilar anastomoses: a persistent trigeminal artery, persistent hypoglossal artery, and a persistent otic artery. These variants should be kept in mind to avoid errors both in clinical reporting and surgical procedures.

## Introduction

During embryological development, various primitive anastomoses occur between the carotid and vertebrobasilar systems (VBS), but only rarely persist into adult life [1,2,3,4,5]. However, if a problem exists during regression, these arteries persist in the adult period and are named as persistent arteries. Some symptoms and clinical problems may be caused by persistent arteries, although they are rare. Knowledge of persistent arteries is very important for diagnosis and treatment. Therefore, characteristic features, diagnostic procedures, differential diagnosis, and clinical significances of these arteries must be known [1]. After a review of the embryogenesis of the VBS, we describe the three different types of persistent anastomoses: trigeminal artery, hypoglossal artery, and persistent otic artery.

## Case Reports

We retrospectively evaluated the images of rare variants of carotid-vertebrobasilar anastomoses that were detected in the patients admitted to our clinic due to various pathologies between 2011 and 2013. The indications for computed tomography (CT) included cerebrovascular disease and vertebrobasilar deficiency. The images were obtained using a 64-slice multiple detector computed tomography (MDCT) (Toshiba Aquilion, 2009, Japan) with the injection of 100 ml of iodinated contrast material at a rate of 4 ml/sec followed by 40 ml saline and a Signa HDxt 1.5T magnetic resonance scanner (USA, 2010) with the injection of 0.05 ml/kg of gadolinium at a rate of 1.8 ml/sec followed by 20 cml saline.

### Case 1

**Figure 1:**
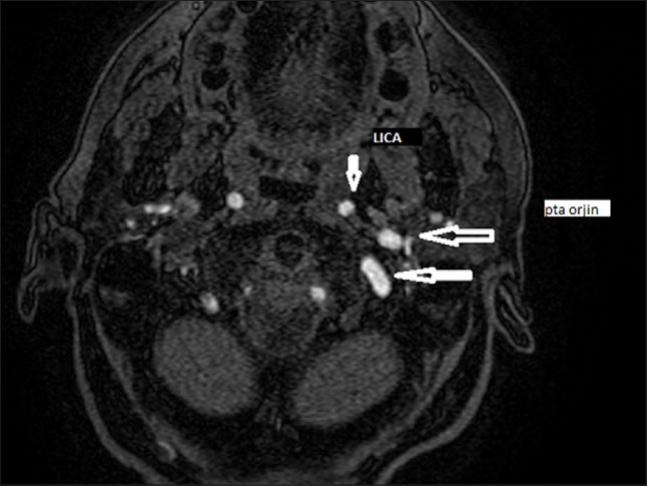
Axial image from thin-section 3D TOF shows the PTA originating from the left internal carotid artery (LICA) (*small arrow*) and duplicating at the same level due to tortuosity (*large arrows*).

**Figure 2:**
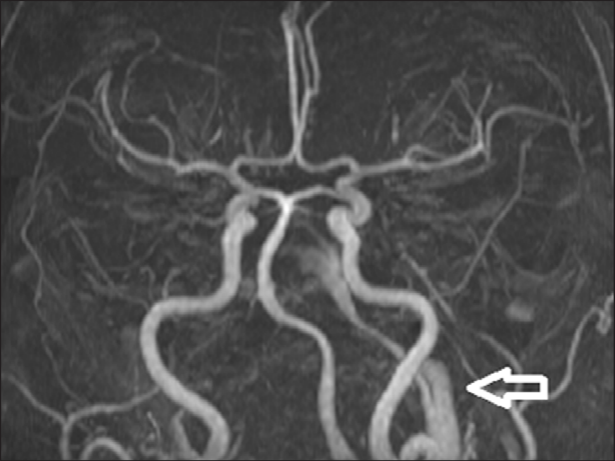
MIP image from 3D TOF MR angiography shows the PTA in the carotid canal on the left side (*arrow*).

**Figure 3:**
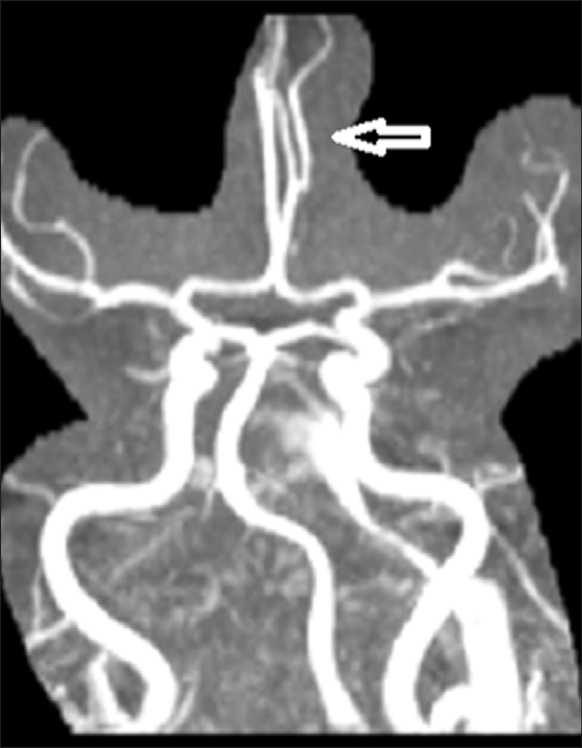
MIP image from 3D TOF MR angiography shows left anterior cerebral artery bifurcation and trifurcation resulting from bifurcation (*arrow*). Early bifurcation and trifurcation at the level of MCA is also seen.

A persistent trigeminal artery (PTA) was rising from the left carotid canal, travelling to the cavernosal segment without joining the basilar artery (BA), and disappearing in the cavernosal segment (Figures 1, 2). In addition, left anterior cerebral artery bifurcation was detected, also causing a trifurcation. The bilateral posterior communicating arteries (PCoA) and the right vertebral artery (VA) were hypoplastic. The middle cerebral artery (MCA) was demonstrating an early bifurcation and trifurcation (Figure 3).

### Case 2

**Figure 4:**
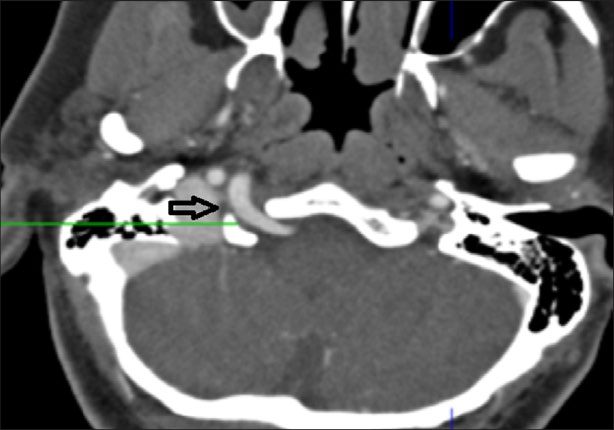
Axial MIP image from 64-slice MDCT shows the PHA passing through the enlarged hypoglossal canal on the right side (*arrow*).

**Figure 5:**
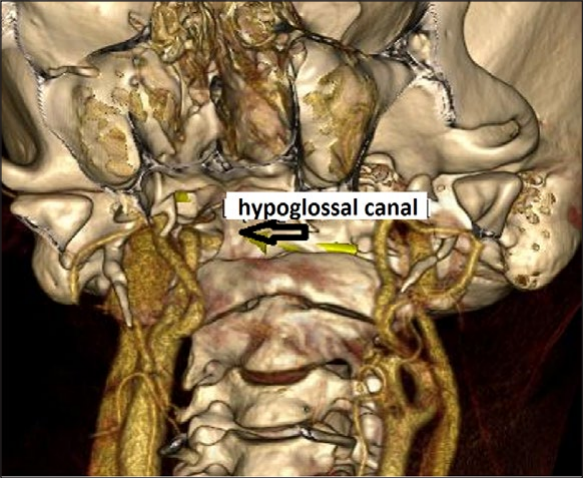
3D volume-rendering image from 64-slice MDCT shows the PHA crossing over the RICA and entering the hypoglossal canal (*arrow*).

**Figure 6:**
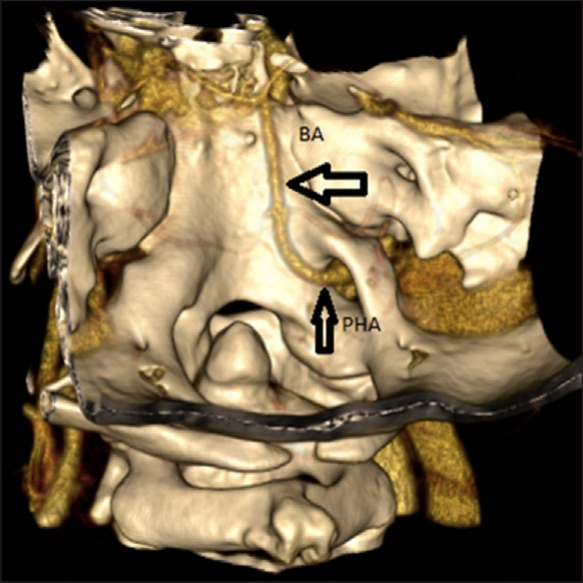
3D volume-rendering image from 64-slice MDCT shows the PHA coursing as the basilar artery (*thick arrow*) after passing through the hypoglossal canal (*thin arrow*). True agenesis of the basilar artery is also seen.

**Figure 7:**
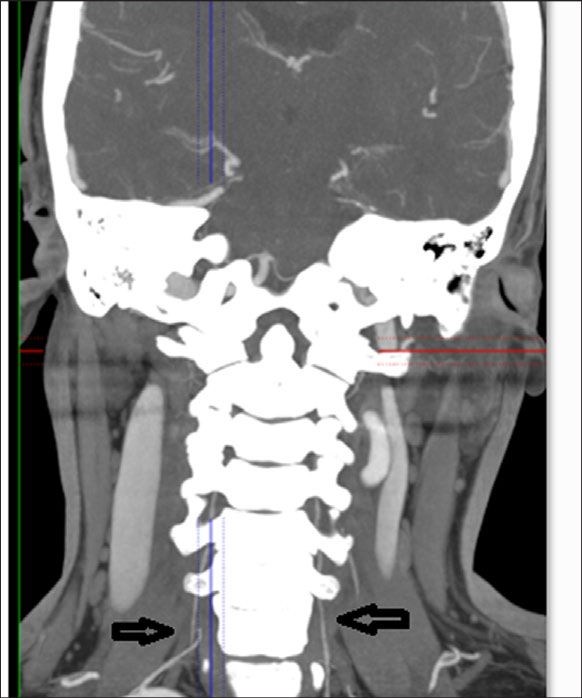
Coronal MIP image from 64-slice MDCT shows severe hypoplasia in the bilateral vertebral arteries in the patient with PHA (Case 2) (*black arrows*).

A persistent hypoglossal artery (PHA) was arising from the right cervical segment of the internal carotid artery (ICA) at the C2 level, passing through the hypoglossal canal, and forming the BA itself (Figures 4, 5, 6). Both of the vertebral arteries were severely hypoplastic and were not forming the BA (Figure 7). The bilateral PCoA were also hypoplastic. The PHA was not joining the BA but was forming the BA itself.

### Case 3

**Figure 8:**
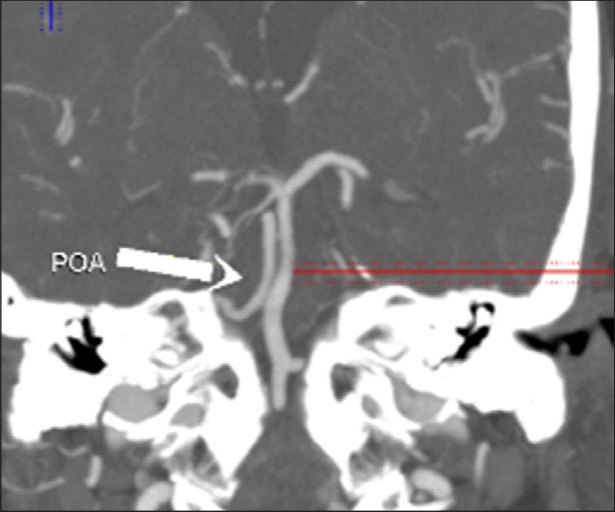
Coronal MIP image from 64-slice MDCT shows the POA exiting from the carotid canal and coursing parallel to the basilar artery (*arrow*).

**Figure 9:**
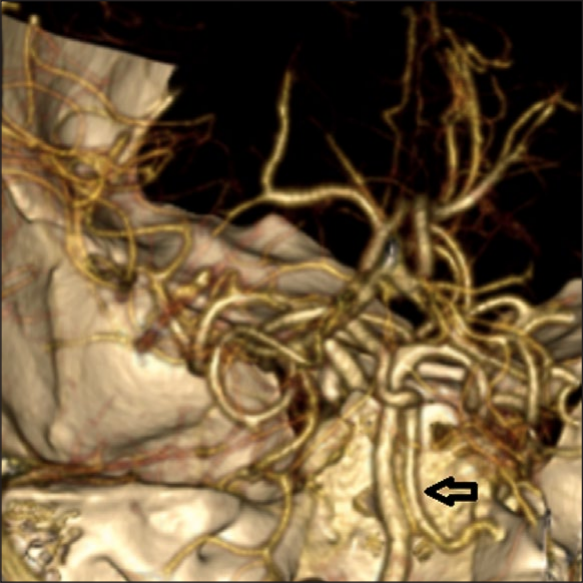
Volume-rendering image from 64-slice MDCT shows the POA coursing parallel to the basilar artery (*arrow*).

**Figure 10:**
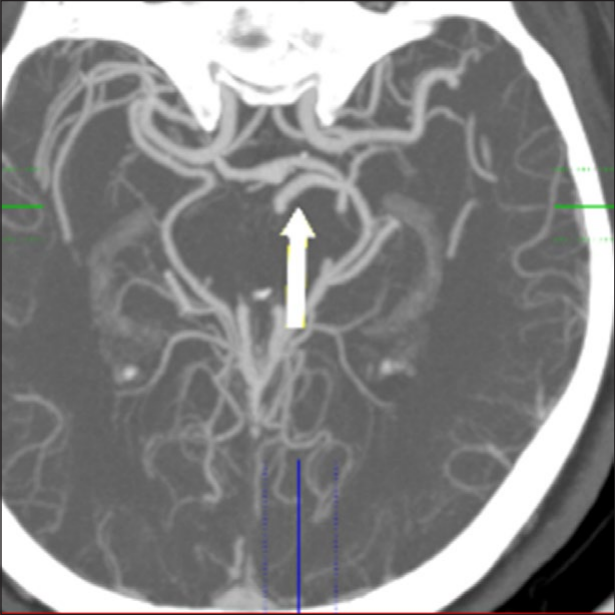
Axial MIP image from 64-slice MDCT shows the POA coursing parallel to the PCA on the anterior side (arrow).

**Figure 11:**
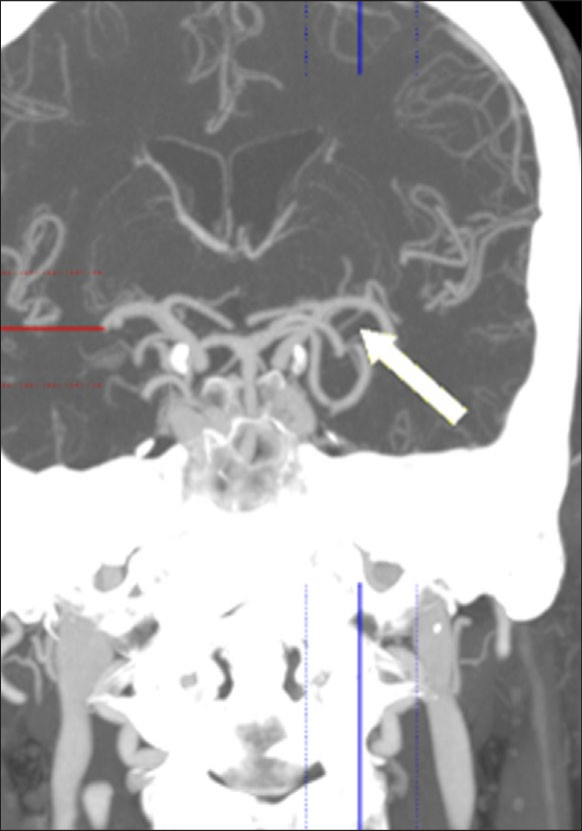
Coronal MIP image from 64-slice MDCT shows the POA joining the M1 segment of the MCA through an accessory PCoA (arrow).

**Figure 12:**
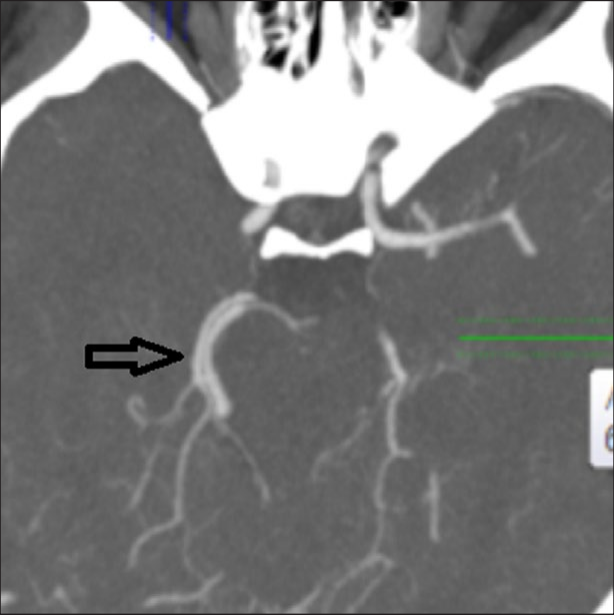
Axial MIP image from 64-slice MDCT shows the hypoplasia in the PCoA and the duplication of the posterior cerebral artery in the patient with POA (Case 3) (arrow).

A persistent otic artery (POA) was rising from the carotid canal and coursing towards the ICA through the right petrous temporal bone (Figures 8, 9). The POA was joining the BA and then coursing towards the left side as a duplication of the posterior cerebral artery (PCA) (Figure 10). POA was joining the M1 segment of the MCA through a communicating artery that originated from the POA (Figure 11). The BA and VA were normal. The bilateral PCoA were hypoplastic (Figure 12). The PCA was localized on the right side, and a branch originating from the PCA was coursing parallel to the M1 segment as a duplication of the MCA.

## Discussion

### Embryology

During the 4-mm to 5-mm embryonic stage, the internal carotid arteries extend through the dorsal aortic arches and anastomoses at three major sites with the longitudinal neural arteries that constitute the primitive vertebrobasilar system. These arteries are named based on their adjacent structures, which include the trigeminal, otic (auditory), hypoglossal, and proatlantal intersegmental arteries. The cervical region involves seven transversely oriented arteries. The first of these, which is also the most cephalic, is the proatlantal intersegmental artery. If any of these vessels fail to disappear during the embryonic stage, various persistent carotid-vertebrobasilar anastomoses may occur [3,5].

Fetal anastomoses occur between the carotid and vertebrobasilar circulations during the 4-mm to 5-mm embryonic stage, persist for approximately one week, and disappear after the development of the vertebral arteries and posterior circulation. These arteries are named based on their adjacent structures: the trigeminal, otic (auditory), hypoglossal, and proatlantal intersegmental arteries. These anastomoses are collectively named “presegmental arteries”. These arteries are seen in the embryonic life, play a key role in the development of the fetal brain, and connect the neural arteries with the internal carotid arteries. The bilateral longitudinal neural arteries later fuse to form the vertebrobasilar system (on the 32nd day of the ovulation, during the 7-mm to 12-mm embryonic stage). The otic artery is the first to disappear, followed by the hypoglossal, trigeminal, and proatlantal intersegmental arteries. These anastomoses may persist after birth and have a reported prevalence of 0.1–1 per cent [3].

During the embryonic stage, the internal carotid arteries extend through the dorsal aortic arches and anastomoses at three major sites with the longitudinal neural arteries that constitute the primitive vertebrobasilar system. These arteries are named based on their adjacent structures. In the craniocaudal order, these arteries are named the trigeminal, otic, hypoglossal, and proatlantal arteries. These arteries often disappear after the formation of the posterior communicating arteries. The otic artery is the first artery to disappear, followed by the hypoglossal, trigeminal, and proatlantal intersegmental artery. However, the primitive carotid-basilar anastomoses rarely persist in adult life. Of these, the persistent trigeminal artery is the most common one, with a reported prevalence of 0.1–0.6 per cent. This artery originates from the ICA immediately following its exit from the carotid canal and anastomoses with the midbasilar artery [1,3,5].

The PTA is categorized into two types, depending on its configuration with the ipsilateral posterior cerebral artery. In the Saltzman type 1, the posterior communicating artery is absent and the persistent trigeminal artery supplies the entire vertebrobasilar system. In the Saltzman type 2, a fetal posterior cerebral artery is present and the ipsilateral P1 segment is absent. In our first case (Case 1), PTA was originating from the left carotid canal, travelling to the cavernosal segment without joining the BA, and disappearing in the cavernosal segment [2].

The PHA is the second most common carotid-vertebrobasilar artery anastomoses, with a reported prevalence of 0.02–0.1 per cent. This artery originates from the ICA at the C1–C3 levels and anastomoses with the BA after passing through the hypoglossal canal [4].

Posterior communicating arteries and vertebral arteries are hypoplastic in 70–80 per cent of cases. In our second case (Case 2), the PHA was originating from the right cervical segment of the ICA at the C2 level, passing through the enlarged hypoglossal canal, and forming the BA itself. Both of the vertebral arteries were severely hypoplastic and were not forming the BA. Moreover, the bilateral PCoA were also hypoplastic, and the PHA was not joining the BA but was forming it itself.

The POA is the rarest form of the presegmental anastomoses. This artery originates from the petrous internal carotid artery within the carotid canal and courses laterally through the internal auditory canal and anastomoses with the proximal BA. In our third case (Case 3), the POA was originating from the carotid canal and coursing distally towards the proximal ICA through the right petrous temporal bone. The POA was joining and travelling with the BA and then coursing towards the left segment as a duplication of the PCA. The POA was joining the M1 segment of the MCA through a communicating artery that originated from the POA itself. This appearance was initially considered as a variant of POA. However, depending on the findings reported in previous studies, we considered that this appearance might be a different variant of PTA.

Some of the arteries developing during the embryonic stage may persist into the adult life, unless they disappear during the intrauterine period. These arteries are named persistent arteries. The anastomoses occurring at the craniovertebral junction (CVJ) are of these types. These anastomoses include the PTA, PHA, POA, and persistent proatlantal segmental arteries. We consider that the variants of carotid-vertebrobasilar anastomoses presented by our patients are important conditions that should be kept in mind to avoid errors both in clinical reporting and surgical procedures.
